# Effect of Electrospun PLGA/Collagen Scaffolds on Cell Adhesion, Viability, and Collagen Release: Potential Applications in Tissue Engineering

**DOI:** 10.3390/polym15051079

**Published:** 2023-02-21

**Authors:** Aldo Guzmán-Soria, Viviana Moreno-Serna, Daniel A. Canales, Claudio García-Herrera, Paula A. Zapata, Pedro A. Orihuela

**Affiliations:** 1 Laboratorio de Inmunología de la Reproducción, Facultad de Química y Biología, Universidad de Santiago de Chile, USACH, Santiago 8320000, Chile; 2 Química y Farmacia, Facultad de Ciencias de la Salud, Universidad Arturo Prat, Casilla 121, Iquique 1100000, Chile; 3 Departamento de Ingeniería Mecánica, Facultad de Ingeniería, Universidad de Santiago de Chile, USACH, Santiago 8320000, Chile; 4 Grupo Polímeros, Departamento de Ciencias del Ambiente, Facultad de Química y Biología, Universidad de Santiago de Chile, USACH, Santiago 8320000, Chile; 5 Centro del Desarrollo para la Nanociencia y Nanotecnología-CEDENNA, Universidad de Santiago de Chile, USACH, Santiago 8320000, Chile

**Keywords:** PLGA, collagen, scaffolds, electrospun fibers, HeLa cells, NIH-3T3 fibroblasts, tissue regeneration

## Abstract

The development of scaffolding obtained by electrospinning is widely used in tissue engineering due to porous and fibrous structures that can mimic the extracellular matrix. In this study, poly (lactic-co-glycolic acid) (PLGA)/collagen fibers were fabricated by electrospinning method and then evaluated in the cell adhesion and viability of human cervical carcinoma HeLa and NIH-3T3 fibroblast for potential application in tissue regeneration. Additionally, collagen release was assessed in NIH-3T3 fibroblasts. The fibrillar morphology of PLGA/collagen fibers was verified by scanning electron microscopy. The fiber diameter decreased in the fibers (PLGA/collagen) up to 0.6 µm. FT-IR spectroscopy and thermal analysis confirmed that both the electrospinning process and the blend with PLGA give structural stability to collagen. Incorporating collagen in the PLGA matrix promotes an increase in the material’s rigidity, showing an increase in the elastic modulus (38%) and tensile strength (70%) compared to pure PLGA. PLGA and PLGA/collagen fibers were found to provide a suitable environment for the adhesion and growth of HeLa and NIH-3T3 cell lines as well as stimulate collagen release. We conclude that these scaffolds could be very effective as biocompatible materials for extracellular matrix regeneration, suggesting their potential applications in tissue bioengineering.

## 1. Introduction

Tissue engineering is a multidisciplinary field that applies the principles of engineering and biology to develop biological substitutes that can be used in the reparation or regeneration of damaged tissue [1]. These substitutes are based on 3D polymeric scaffolds containing a porous and interconnected structure that can mimic the extracellular matrix (ECM) and act as temporal support for tissue regeneration [2]. In this sense, the scaffolds processing technique and the biomaterial used for its manufacture are crucial aspects to consider. Therefore, electrospinning is a simple and versatile technique to obtain scaffolds with fibrous 3D structure, high porous and pore interconnectivity, and high surface area morphology, similar to the ECM structure, allowing the transport of nutrients and regulating the signaling pathways responsible for cell absorption, proliferation, and differentiation [3].

On the other hand, an adequate strategy to develop scaffolds is combining a synthetic polymeric and a natural polymer, thus assuring a biomaterial with excellent mechanical properties, biodegradation, and biocompatibility [4]. Within the synthetic polymers used to obtain scaffolds, poly(lactic-co-glycolic acid) (PLGA) has great potential because it is a biodegradable and biocompatible polymer, is approved by the Food and Drug Administration (FDA) to be used in the human body, so it has been used in various biomedical applications as well as implants and controlled release of drugs [5]. In addition, the PLGA has the advantages of being easy to process, having excellent mechanical properties, and controlled degradation, which makes this polymer an alternative for regenerating tissue since this polymer can be biodegraded at the same time new tissue is being generated [6,7]. Although PLGA has been used as absorbable sutures, its degradation rate and compounds as lactic acid and glycolic acid can be customized to suit its application [8]. However, the hydrophobicity of PLGA and lack of cell affinity by the absence of surface cell recognition sites limits cell adhesion and, thus, its use as scaffolds in tissue engineering. Therefore, PLGA could be mixed with natural polymers such as collagen. Collagen is one protein found in different tissues currently, 20 families have been described, and the most abundant is type I collagen, which is present in bone tissue, skin, tendons, and ligaments [9]. Collagen has a complex hierarchical configuration that can be divided into a primary structure (amino acid triplet), secondary structure (α-helix), tertiary structure (triple helix), and quaternary structure (fibrils) [10]. In addition, collagen with a fiber size of 1 μm has an excellent binding capacity to transmembrane receptors located in the cell, such as integrins α1β1, α2β1, α10β1, and α11β1 and domain receivers discoidine (DDRs), by a lot can form anchors that allow better cell adhesion [11].

Some reports have indicated the capacity of PLGA to be used as an ideal support for extracellular matrix (ECM) regeneration [12,13]. For example, Shin et al. obtained electrospun PLGA scaffolds for their application in cartilage regeneration. They obtained the scaffolds from three types of lactic acid and glycolic acid content ratios in PLGA 75:25, PLGA 50:50, and a mixture of both relations. In addition, the cytotoxicity, cell proliferation, and the formation of a new ECM in porcine articular cartilage chondrocytes were evaluated. As a result, it was found that the tensile modulus and ultimate tensile stress were higher as the content of lactic acid increased in the PLGA (PLGA 75: 25), 110.8 ± 10.1 MPa and 4.67 ± 0.31 MPa, respectively. Furthermore, tensile deformation of the mixture presented a value higher of 173.7 ± 8.7% compared to PLGA 75:25 and PLGA 50:50. Moreover, it was observed that the chondrocyte cells adhere to the PLGA scaffolds, do not generate cytotoxicity, increase cell proliferation, and the formation of a new ECM that is found on the first day with 7 ng of glycosaminoglycans (GAG) and it increases at 14 days to 13 ng of GAG, suggesting the formation of new extracellular matrix [14]. Chen et al. found that collagen in mesh PLGA increases the growth of fibroblasts in the mesh more quickly and permits forming of a more uniform layer of dermal tissue than in the PLGA mesh without collagen. Besides, dermal tissue was formed after 2 weeks and became epithelialized after 4 weeks in the presence of collagen [15].

Also, Liu et al. obtained electrospun PLGA/collagen scaffolds in different relations (100/0, 80/20, 65/35, 50/50, 0/100, *v*/*v*) to be used as wound dressings. PLGA/collagen presented a fiber diameter of 170 nm to 650 nm, and the fiber diameter decreased up to 190 nm with the collagen content compared to PLGA fibers (460 nm). The wettability of the scaffolds increased by ca. 200% with higher collagen contents, which favored cell proliferation after 14 days, as was observed by the MTT assay. Furthermore, in the wound healing trial, authors found that the area covered by electrospun PLGA/collagen scaffolds is reduced to 5% at 21 days, so the researchers concluded that these scaffolds might be suitable for use as dressings for skin wounds [13].

Brown et al. developed a PLGA/collagen scaffold with a highly porous structure. In this case, PLGA nanofibers with a size fiber of 0.9 µm were fabricated by electrospinning and then were superficially and chemically modified with type I collagen (4.4 ± 0.3 µg/mg). Scaffolds in the presence of collagen showed an increase in the synthetic activity of primary human hepatocytes, and the viability was maintained for 14 days. Besides, aggregates of hepatocytes were spreading along and across the nanofibers of PLGA/collagen but were absent in unmodified PLGA scaffolds. This study demonstrated that collagen in scaffolds could stimulate long-term in vitro survival of primary hepatocytes [16].

The studies mentioned above reinforce the strategy to use fibers of PLGA/collagen as scaffolds for tissue regeneration. For this reason, we wanted to expand the studies of these PLGA/collagen scaffolds as supports for cell adhesion and viability, mainly of the HeLa epithelial cells, which are the main components for extracellular matrix formation and tissue regeneration [17].

Finally, another aspect to consider in the use of PLGA for biomedical application as tissue engineering, wound dressing, and drug delivery is a molar relation between lactic acid (LA) and glycolic acid (GA) monomers, being the more typical relations (75/25) and (50/50) [18]. On the other side, when the GA monomer increases in the copolymer composition, the degradation rate is faster [19]. Shin et al. reported the preparation of electrospun fibers based on PLGA (75/25) and (50/50) for articular cartilage reconstruction. They found that the mechanical properties and degradation process are modulated by the molar ratio of (LA/GA) to a major quantity of GA monomer, increasing the hydrophilic character and the degradation rate [14]. Hong et al. develop electrospun fibers based on PLGA (50/50) containing rifampin as an antibiotic for wound dressing applications. Their results indicate that the degradation process reaches a 60 wt.% after 10 days, which is suitable for these kinds of applications [20].

Therefore, the objective of this research was to develop biocompatible scaffolds based on a mixture of PLGA with a molar ratio of 50:50 of lactic and glycolic monomers and type I collagen by electrospinning with potential applications for tissue engineering. First, PLGA and PLGA/collagen fibers were obtained using the electrospinning technique. Then, the fibers were characterized structurally and morphologically, and thermal and mechanical properties were evaluated. The capacity of the scaffolds to induce ECM regeneration was also explored by cell adhesion, viability, and collagen release assays in the human cervical adenocarcinoma cell line HeLa and NIH-3T3 fibroblasts isolated from an NIH/Swiss mouse embryo.

## 2. Materials and Methods

### 2.1. Materials and Reagents

Poly (lactic-co-glycolic acid) (PLGA) (ratio of 50:50, RG 504, Mw = 38,000–54,000 g/mol) (Darmstadt, Germany), lyophilized rat tail type 1 collagen (Mannheim, Germany), MTT (3-[4,5-dimethylthiazol-2-yl]-2,5 diphenyl tetrazolium bromide) (Darmstadt, Germany) and 1,1,1,3,3,3-hexafluoro-2-propanol (HFIP) (Milan, Italy).

### 2.2. Preparation of PLGA and PLGA/Collagen Scaffolds Using the Electrospinning Technique

PLGA and PLGA/collagen scaffolds were obtained by electrospinning equipment Tong li Tech, TL-01 (Shenzhen, China) following a previously reported procedure [21,22]. First, PLGA and collagen dissolved into HFIP at 25% and 5% (*w*/*v*) concentrations, respectively. The PLGA and collagen solutions were then mixed in a 90:10 ratio to obtain a PLGA/collagen solution that was stirred for 36 h. The previously prepared PLGA and PLGA/collagen solutions were deposited in a 2 mL plastic syringe with a needle of diameter 21 G and then attached to the infusion pump. The parameters of the electrospinning process were standardized to obtain the fibers, which correspond to an infusion rate of 1 mL/h, a voltage of 12 kV, the distance between the needle and the collector was 12 cm, and the process was carried out at room temperature. The collected fibers with a final concentration of 2 wt% of collagen were stored under refrigeration

### 2.3. Characterization of PLGA and PLGA/Collagen Scaffolds

#### 2.3.1. Morphological Analysis

The morphology of the scaffolds was analyzed by scanning electron microscopy (SEM) (SEM EVO IMA 10 Zeiss, Jena, Germany) at an acceleration voltage of 10 kV. The samples were coated with gold by spraying at a current of 30 mA and placed on the sample holder of the SEM equipment. The images obtained from the SEM were analyzed with the Image J software to determine the size of the fiber, and 50 measurements were considered in different fibers selected at random.

#### 2.3.2. Characterization by ATR-FTIR Spectroscopy

An analysis of the chemical structure of the scaffolds of PLGA and PLGA/collagen was conducted by Fourier transform infrared spectroscopy with attenuated total reflectance (ATR-FTIR, Spectrum Two, Perkin Elmer, Llantrisant, UK), and the bands were analyzed in a frequency range between 400–4000 cm⁻^1^. As a reference, lyophilized rat tail type 1 collagen was used. Besides, in order to verify that the structural integrity of the collagen was maintained after contact with the solvent, a collagen film was prepared by the solvent casting method. For this purpose, the first 30 mg of collagen was mixed in 600 μL HFIP with stirring and left in contact for 6 h, and then the solvent was evaporated for 24 h in an oven at 40 °C.

#### 2.3.3. Contact Angle Measurements

Measurements of static contact angle were performed on a computer Dataphysics O.C.A. 20 (Filderstadt -Germany) to obtain the hydrophilic properties of the scaffolds. For this, 10 mm × 10 mm squares of samples were cut and prepared. After 10 μL of distilled water, (a drop) was placed on the surface of the fibers. The test was performed 3 times, and the average was reported.

#### 2.3.4. Enzymatic Degradation Test

The degradability of the scaffolds was analyzed quantitatively by weight loss with an enzymatic degradation test. First, the scaffolds were cut into sizes of 1.0 × 0.6 cm, a thickness of 0.10 mm, and a weight between 10 to 11 mg. Then, scaffolds were immersed, by triplication, in a PBS (pH 7.4) solution with 60 μg/mL collagenase. The tubes were placed inside an incubator with a shaker at 37 °C for a total period of 7 days. The scaffold samples were removed in 1, 4, and 7 days. Next, samples were washed with EDTA, and distilled water, dried in an oven for 2 weeks at 37 °C, and finally transferred to a desiccator. The percent weight loss was calculated according to the following equation:

(1) $$Weight\;loss\;\left(\%\right)=\frac{W_{0}-W_{Dt}}{W_{0}}\;.$$

where *W*_0_ is the initial weight of the sample before the enzymatic degradability test, and *W_Dt_* corresponds to the weight of the dry sample after the corresponding time of degradation (1, 4, and 7 days).

After the samples were dried, they were analyzed using ATR-FTIR spectroscopy and scanning electron microscopy (SEM).

#### 2.3.5. Thermal Properties and XRD Diffraction

The glass transition temperature (Tg) of the PLGA and denaturation temperature (Td) of the collagen were measured by differential scanning calorimetry (DSC) (Mettler Toledo DSC-1 starter system, Columbus, OH, USA). 4.5–5.5 mg of each sample were weighed. The samples were heated in a temperature range of −30 °C to 200 °C with a heating rate of 10 °C/min. As a reference, lyophilized rat tail type 1 collagen was used. We also had to verify that the structural integrity of the collagen was maintained after contact with the solvent, as described above.

The crystallographic structure of polymers was analyzed using an X-ray diffractometer. The study was carried out in a Siemens D5000 diffractometer, using 0° < θ° < 80° scanning.

#### 2.3.6. Mechanical Properties of Fibers

The mechanical properties of the PLGA and PLGA/collagen scaffolds were analyzed by uniaxial traction equipment (Cell Scale Biomaterials testing, Waterloo, Canada). First, the scaffolds were cut into strips of (18 × 3 mm). Then, the samples were lengthened to a tractive speed of 5 mm/min with a displacement ramp of 60,000 microns and a duration time of 720 s. This test was performed in triplicate to define average values and standard deviations, and stress-strain curves were used to determine Young’s modulus, tensile strength, and elongation at break.

### 2.4. Cell Culture

HeLa and NIH-3T3 cell lines (gently donated by Dr. Gareth Owen from Pontificia Universidad Católica de Chile and Dr. Kevin Maisey from Universidad de Santiago de Chile) were used to study cell adhesion and viability. These cells were cultured in DMEM medium with 100 U/mL of penicillin, 1 μg/mL of streptomycin, and 0.25 μg/mL of fungizone with 10% fetal bovine serum (FBS) for the cell adhesion assay and 5% *v/v* of fetal bovine serum (FBS) for the cell viability assay with an atmosphere of 5% CO_2_ and air at 37 °C [23].

### 2.5. Cell Adhesion and Viability

The PLGA and PLGA/collagen scaffolds were sterilized with ultraviolet light for 20 min on each side, then hydrated with PBS for 8 h, and the cells were also hydrated for 16 to 24 h. During that time, the cells took time to adhere to the scaffolds, resulting in a reduction in hydrophobicity [12]. HeLa cells and NIH-3T3 fibroblasts were seeded on separately electrospinning scaffolds at a cell density of 50,000 cells in 24-well plates for 48 h. After this time of cell culture, the plates were washed twice with PBS to remove the cells that had not adhered to the scaffolds. The cells were then fixed with a 2.5% glutaraldehyde solution for 1 h. Subsequently, they were dehydrated for 10 min in different concentrations of ethanol (20, 40, 60, 80, 90, and 100% *v*/*v*). Cell morphology was analyzed by scanning electron microscopy (SEM, EVO IMA 10 Zeiss, at an acceleration voltage of 10 kV). The images obtained were analyzed using Image J software.

Cell viability in the scaffolds was studied by the MTT assay. This method is based on the reduction in bromide from 4,5-dimethylthiazole-2-yl-2,5-diphenyltetrazole (MTT) by the mitochondrial enzyme succinate dehydrogenase. As reported by Boncler et al. [24] this test has a lower inter-assay variability and a signal-noise ratio, which makes it more useful for our study. Furthermore, according to the manufacturer, the MTT assay is more useful for experiments requiring longer procedure times, as used in our cell viability experiments.

The scaffolds were sterilized with ultraviolet light for 20 min on each side and hydrated for 24 h. Next, HeLa cells and NIH-3T3 fibroblasts were seeded at a cell density of 4000 cells separately on electrospun scaffolds in 96-well plates for 1, 3, 5, and 7 days. After these times, the culture medium was removed and replaced by 100 μL of DMEM medium mixed with 50 μL of MTT (5 mg/mL) to be incubated for 4 h at 37 °C. Then 30 μL of culture medium was left in the well, and 100 μL of DMSO was added to help dissolve the formazan crystals. Finally, the multiwall plates were placed in the sample holder of the spectrophotometer (Tecan infinite F50) to read its absorbance at 570 nm. The assays were performed in triplicate, and a positive control based on only cells on plates and collagen hydrogel as a 3D control was used for comparison [25].

### 2.6. Collagen Release Assay

The release of collagen by fibroblasts NIH-3T3 was quantified in the culture medium using the Collagen Assay Kit (Sigma Aldrich, St. Louis, MO, USA). This kit is based on the enzymatic digestion of collagen to obtain peptides with the N-terminal end that respond to the reactive dye to form a fluorescent complex measured at an intensity of 375/465 nm. Thus, 2000 cells/well were cultured on PLGA and PLGA/Collagen scaffolds in 96-well plates for 5 days, and then the culture medium was harvested to determine the collagen concentration as described above. PLGA/collagen scaffolds incubated without cells were used as a control group [26].

### 2.7. Statistical Analysis

The experimental data are shown as the mean and standard deviation. In addition, a statistical analysis of the data was performed by the analysis of variance (ANOVA) with the GraphPad Pris 8 program, and the statistically significant differences were considered when the *p*-value < 0.05.

## 3. Results and Discussion

### 3.1. Morphological Analysis

**Figure 1:**
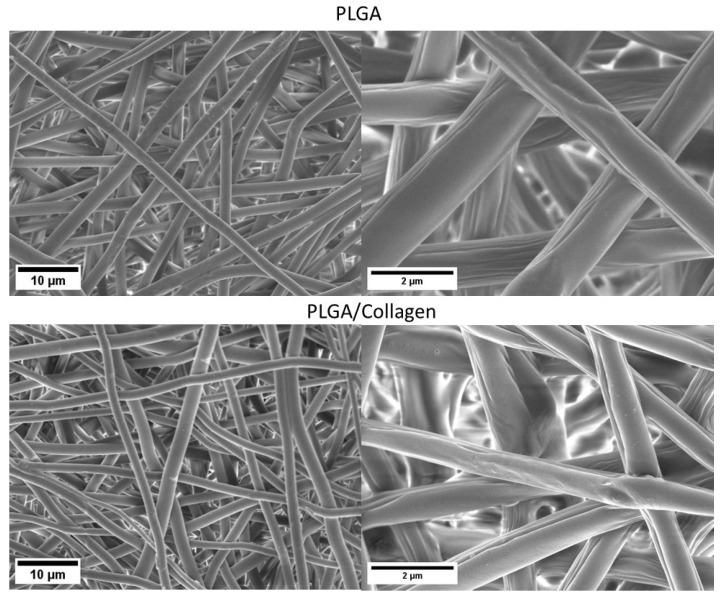
SEM images of PLGA and PLGA/collagen scaffolds.

**Figure 2:**
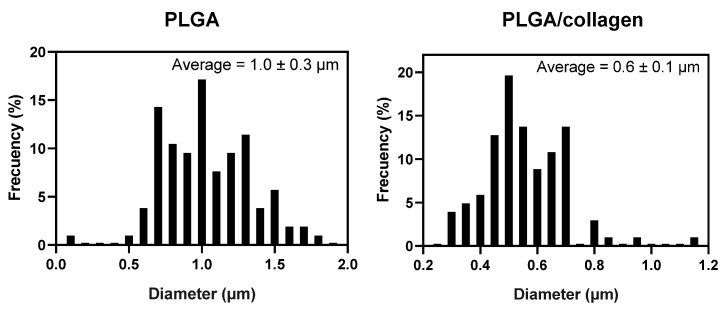
Fiber diameter histograms of corresponding PLGA and PLGA/collagen scaffolds.

Figure 1 shows the SEM images of the PLGA and PLGA/collagen fibers obtained by the electrospinning method. For both samples, a 3D three-dimensional structure with high porosity and pore interconnectivity was obtained. The fibers had a random arrangement and a homogeneous surface free of defects. This suggests an efficient electrospinning process under optimal blending conditions of PLGA and collagen. In Figure 2, the histograms of fiber diameter distribution, in both cases, a bimodal-type distribution was obtained, finding two groups of diameters that prevail over the rest. Quan et al. reported that electrospun fibers with a bimodal distribution show a better pore structure, improving cell adhesion, and better hydrophilicity and tensile properties [27]. The diameter average of fiber for the PLGA scaffold was 1.0 ± 0.30 μm while the PLGA/collagen scaffold was 0.6 μm ± 0.1 μm. Zhao et al. reported the preparation of PLGA electrospun fibers with different concentrations between 0.15 and 3.0 (g/mL) in a mixture of DMF and THF as a solvent. When the concentration was 1.5 (g/mL), the average diameter was 100 to 200 nm; however, for values between 2.5 and 3.0, the average increased in the 400 to 1000 nm range and showed a bimodal distribution [28]. Deitzel et al. reported the preparation of electrospun fibers based on poly(ethylene oxide) PEO on different concentrations ranging from 7 to 20 wt.%, finding that when the concentration was in the upper 15%, the electrospun fibers showed a bimodal distribution related mainly to the concentration and the droplet ratio, causing the in-flight splitting effect during the electrospinning process [29].

On the other hand, the incorporation of collagen decreased the average diameter by ca. 46%. This result suggests that the reduction can be related to the change in the conductivity properties of the solution. Collagen contains amide groups in its structural conformation, which generates an increase in solution conductivity during the electrospinning process, making the jet trajectory longer and inducing further stretching of the solution [30]. Kwon et al. found that fiber diameter was reduced in electrospun blends of PLCL (poly (L-lactide-co-3-caprolactone)) and collagen. The fiber diameter decreased from 500 nm to 450 nm by incorporating as little as 5 wt% collagen. The authors attributed this result to the specific electrical properties of collagen compared to the neat polymer [31]. Similar results were reported by other authors who studied the obtention of PLGA/collagen fibers using different relations: 100/0, 80/20, 65/35, and 50/50, 0/100 by electrospinning. The fibers also showed a decrease in diameter with the collagen content, from 460 nm for 100% PLGA to 190 nm for 100% collagen [13]. The same behavior was found when chitosan (CS) was incorporated into the polycaprolactone (PCL) by the electrospinning method. The fibers of neat PCL showed a diameter of 212 ± 25 nm, and the presence of the CS decreased the diameter to 186 ± 18 nm. The authors explained that this occurred because CS increased the polarity of the electrospinning solution due to its charged functional groups, producing the stretching of fibers under an electrical field. The author also highlights that the ZnO incorporation into PCL/CS increased the solution’s conductivity and caused a decrease in fiber diameter [32].

### 3.2. Characterization by ATR-FTIR Spectroscopy

**Figure 3:**
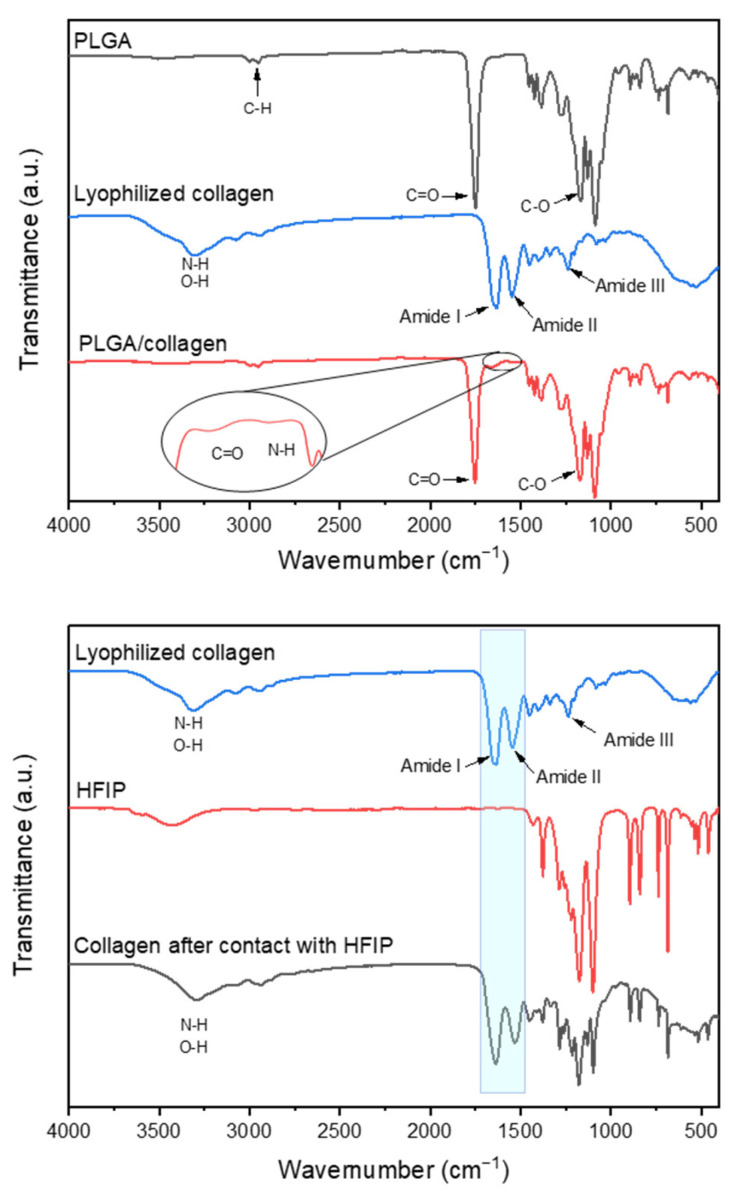
Fourier transform infrared spectroscopy (FTIR) spectra of PLGA, PLGA/collagen scaffolds, lyophilized collagen, and collagen prepared by solvent casting in HFIP.

Figure 3 shows the spectra of PLGA, PLGA/collagen fibers, and lyophilized type I collagen. PLGA fibers spectrum showed low-intensity signals at 2990 and 2953 cm^−1^, which are attributed to asymmetric stretching of the C−H bond of the −CH_2_− and −CH− groups of the aliphatic chain of PLGA, respectively. The intense signal at 1757 cm^−1^ is attributed to the C=O stretching of the ester, and the signals at 1170 and 1076 cm^−1^ correspond to the C−O single bond stretching of the ester group [33]. Type I collagen shows a characteristic FT-IR spectrum with the broad signal at 3314 cm^−1^ attributed to the stretching O−H and N−H. Low-intensity signals in the range of 3076 and 2928 cm^−1^ are assigned to the C-H stretching of the aliphatic amino acids in the collagen protein [34]. The absorption signal at 1640 cm^−1^ is representative of amide I by stretching vibrations of peptide carbonyl group C=O groups [35]. The signal at 1550 cm^−1^ is attributed to amide II due to N−H bending, and the weaker band centered at 1242 cm^−1^ represents amide III vibration, which constitutes a contribution from the N−H in-plane deformation [36]. The FT-IR of PLGA/collagen spectra did not reveal any significant difference between the functional groups of PLGA fibers due to the higher concentration of the last one in the fibers. However, low-intensity signals at 1640 and 1550 cm^−1^ due to amides I and II confirm the successful incorporation of collagen onto the scaffolds without losing its structural nature during the electrospinning process.

In order to confirm the denaturation of collagen in the presence of HFIP solvent in the electrospinning process, an FTIR spectrum of the collagen prepared by solvent casting was collected. As seen in the spectra, the intensity of the signals contributed by the amide I and II of collagen are maintained after contact with the solvent, indicating indicated the higher degree of molecular order of the collagen [36]. Due to the presence of signals corresponding to traces of solvent in the film in 1500 to 1000 cm^−1^ region, the peak attributed to amide III cannot be appreciated; however, the band around 1454 cm^−1^ due to the bending mode of the C-H bond can be observed. According to some reports, the presence of this last peak also suggests that the triple helical structure of collagen is maintained [34,37,38,39]. This confirms that there is no loss of the collagen structural nature in the presence of HFIP solvent, so the electrospinning process should not be affected either. Although HFIP is an organic solvent that could lead the protein denaturation, it has been reported by Hall Barrientos et al. that the use of this solvent can be very important for the formation of PLA/collagen fibers due to its low boiling point (58.2 °C) that quickly allows evaporation during the electrospinning process [40]. Besides, the high affinity of collagen with the solvent could increase the solution conductivity during the electrospinning process, making the jet trajectory longer and inducing further stretching of the solution [41].

### 3.3. Contact Angle

The wettability of the surface of the scaffolds is crucial because it ensures an adequate biological response, especially a desirable adhesion cell [42]. Contact angle measurement estimates the wettability of a biomaterial and corresponds to the ease of diffusion of liquid on the surface; besides, it depends principally on variables such as surface roughness/topography and surface tension [43]. Therefore, the lower contact angle in water can be interpreted as a scaffold with a higher degree of hydrophilicity [44]. The value for PLGA fibers was 136.5°, indicating the polymer’s hydrophobic nature near the value reported by Sadeghi et al., who obtained an angle of 132.6° for PLGA nanofibers [45]. A slight reduction in the contact angle for PLGA/collagen fibers to 132.6° was observed after 10 min of exposure to a water drop, which can be due to the low amount (ca. 2.0 wt%) of collagen in the blends with PLGA. Although the incorporation of collagen, even in low amounts, enhances the hydrophilic properties of scaffolds, the hydrophobic property is strongly dominated by the presence of PLGA. Mozafari et al. indicated that the hydrophilicity of PCL/collagen electrospun scaffolds is proportional to the collagen content of scaffolds. Thus, scaffolds containing higher amounts of collagen could present a low contact angle and were more hydrophilic [46].

### 3.4. Enzymatic Degradation Test

**Figure 4:**
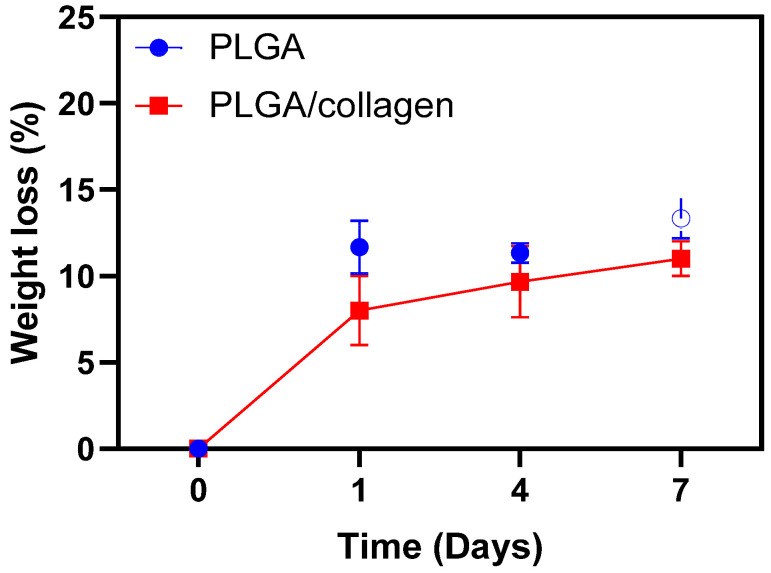
Weight loss of PLGA and PLGA/collagen scaffolds in the time exposed to enzymatic degradation with collagenase.

The extracellular matrix is a highly dynamic structure that constantly rebuilds and models to maintain cell homeostasis over all tissues [47,48]. In this context, scaffolds should be biodegradable gradually over a certain period to provide enough space for cell growth until the tissue is entirely regenerated [4]. Therefore, the biological stability of PLGA and PLGA/collagen scaffolds was measured via degradation against collagenase enzyme for 7 days, and Figure 4 presents the results of the scaffolds degradation test. The degradation of the scaffolds is presented as the percentage of weight remaining after exposure to an enzymatic solution containing collagenase. Both PLGA and PLGA/collagen scaffolds present a weight loss of approximately 10% on the first day, and then this percentage was maintained for 7 days. However, the degradation of PLGA was slightly higher than the PLGA/collagen scaffold reaching a 4% more degradability. Thus, the weight loss indicates that during 7 days the process of degradation of the fibers was slow. Therefore, the results could be related to the interactions between collagen and PLGA, conferring stability to both. This means that in 7 days, collagen inside of fiber cannot interact with collagenase [49]. This same behavior has been observed by Said et al., who observed that PLGA (ratio of 50:50) fibers presented stability to the degradation in PBS during 10 days [50].

Sadeghi et al., who obtained electrospun scaffolds of PLGA coated with collagen, observed that the degradation of both PLGA and PLGA/collagen scaffolds is nearly similar. However, the weight loss of the coated collagen fibers was approximately 4.2% after 1 week, and for the non-coated fibers, it was 3.2% [45]. The authors explained that due to the low amount of collagen (ca. 2 mg/mL) in the composite, its degradation percentage is negligible compared to PLGA degradation, and the difference between both scaffolds is not significative.

**Figure 5:**
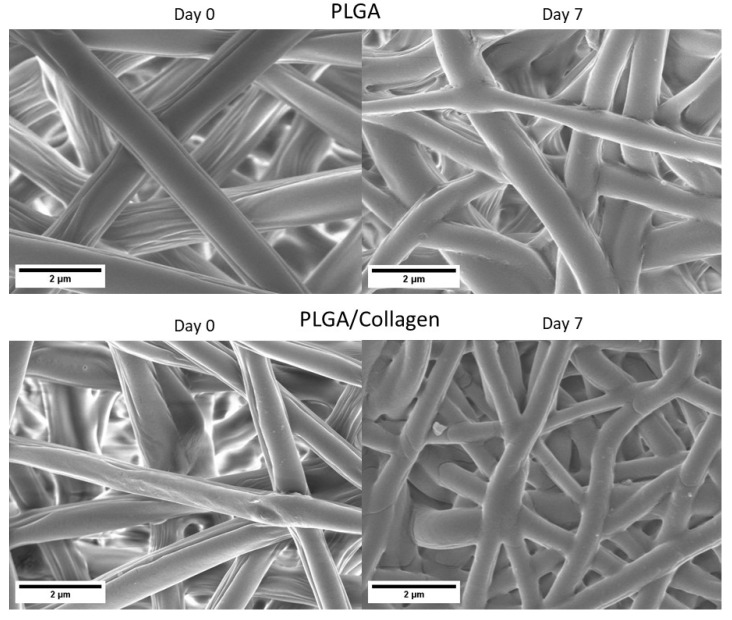
SEM images of enzymatic degradation of PLGA and PLGA/collagen scaffolds after 7 days of exposure to PBS and collagenase.

Through SEM analysis (Figure 5), it was possible to corroborate that the PLGA and PLGA/collagen scaffolds retained their integral porous structure after 7 days in PBS solution and collagenase. No signs of fractionation of the material are appreciated. This indicates that at the time of the study, the material continues to be good support for cell adhesion and guides regenerative processes. However, it is possible to observe that the fiber diameter increased from 1.0 ± 0.3 to 1.6 ± 0.4 μm for PLGA and from 0.6 ± 0.1 to 1.1 ± 0.4 μm for PLGA/collagen, which is mainly attributed to a swelling process of the fibers upon contact with the PBS solution. Sadeghi-avalshahr et al. reported that the degradation of fibers based on PLGA (75:25) (Lac:Glic) and PLGA/collagen have a degradation of 29% after 4 weeks, noting that most of the mass loss occurs in the first week, attributing the fast degradability to the presence of collagen. Usually, the degradation rate is low due to the majority proportion of lactic monomer (75), which gives it more hydrophobicity [12]. Considering these results and considering that the PLGA used has a ratio of lactic and glycolic monomers of 50:50, increasing the hydrophilic character, they could explain this swelling phenomenon [12].

**Figure 6:**
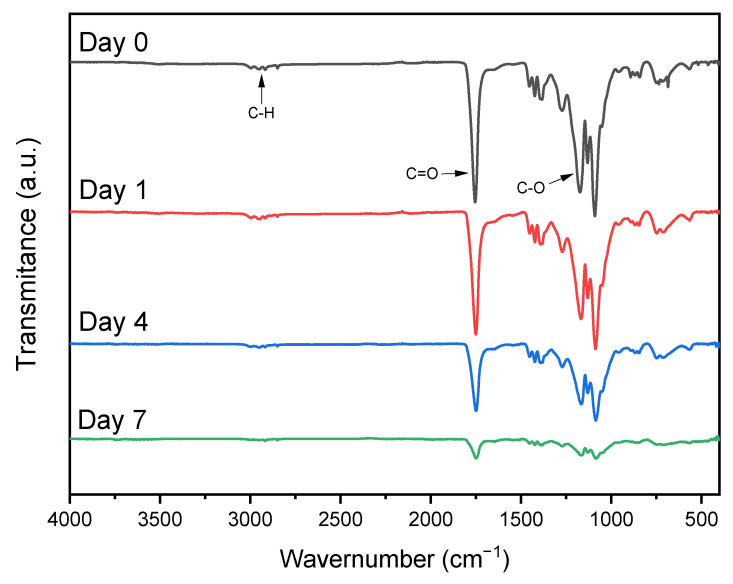
FTIR spectra of PLGA/collagen scaffolds until complete 7 days of degradation in collagenase.

In order to understand the chemical behavior of degradation on the surface of the PLGA/collagen fibers, ATR-FTIR spectra were collected each time for up to 7 days in the presence of collagenase (Figure 6). As observed in Figure 6, a strong decrease in the peaks corresponding to amides I and II of collagen was observed by day 7, indicating a surface degradation of the collagen component of the fiber with the contact of collagenase [51]. A remarkable decrease in the peak intensity was also noted in the C=O stretching of carbonyl from the ester and C-O present in PLGA. This demonstrates that by 7 days, the surface of fibers shows the first signs of degradation. However, it is not enough time to complete the degradation of the scaffolds.

Some authors have explained the degradation mechanism of PLGA. The presence of ester linkages in the polymer backbone allows gradual hydrolytic degradation. The degradation products are their corresponding monomers, lactic and glycolic acids, which are non-toxic. The degradation mechanism of PLGA consists of two steps: the first step of degradation is hydration which is when water enters the amorphous region, and hydrogen bonds are broken. Then the initial degradation phase is where the breaking of covalent bonds and a decrease in molecular weight occurs, followed by constant degradation, where the massive cleavage of the covalent bonds occurs [52,53].

Therefore, based on the results of this study, we can assure that the scaffolds obtained in this study could be appropriate for tissue regeneration due to the resistance of the scaffolds in the enzymatic degradation process during 1 week.

### 3.5. Thermal Properties and XRD Analysis

**Figure 7:**
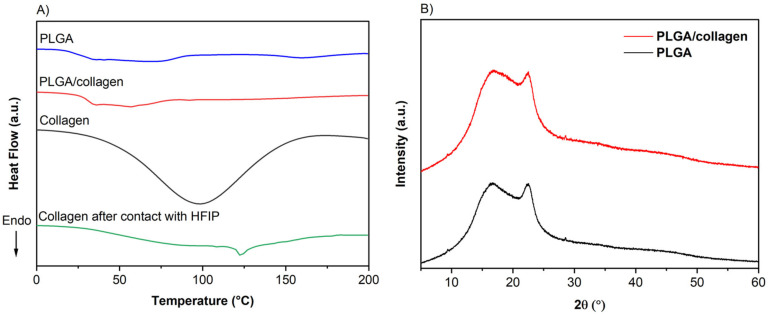
(**A**) Differential scanning calorimetry (DSC), and (**B**) XRD pattern of PLGA and PLGA/collagen. Thermograms of lyophilized and prepared by solvent casting in HFIP collagen were also shown for comparison.

Figure 7A displays the DSC thermograms of PLGA and PLGA/collagen scaffolds compared to lyophilized collagen. The glass transition temperature (Tg) and denaturation temperature (Td) were obtained from the first heating. The thermograms indicate the amorphous principally nature of PLGA with a Tg at 33°C, similar to the reported in the literature for PLGA fibers obtained by the electrospinning method [12,42]. Besides, it can be observed an endothermic peak at 56 °C, which other authors have associated with the enthalpic relaxation of PLGA, characteristic of raw PLGA [30,54,55].

Fouad et al. prepared PLGA fibers using copolymer with L/G ratio 75:25 and compared them with bulk PLGA [56]. The DSC thermogram showed the amorphous nature of PLGA with a glass transition temperature (Tg) around 55 °C and 51 °C for bulk PLGA and PLGA nanofibers, respectively. Similar results were found by Liu F et al., where fibers were obtained using different molecular weights of PLGA (Mw: 15,000, 45,000, and 81,000 g.mol-1) and compared with the raw PLA [55]. The authors discuss that the fiber structure’s crystallinity decreased appreciably compared to the raw PLGA polymer; therefore, the Tg also decreased. Furthermore, the chain entanglement in bulk form is much higher when compared to the same polymer in nanofiber form, decreasing their crystallinity [55,56]. This could be due to the improvement in the orientation of molecular chains in the electrospun polymer nanofibers and the larger area-to-volume ratio of electrospun fibers [55]. On the other hand, Silva et al., and LiH et al., synthesized PLGA and used PLGA (LA : GA : 75 : 25), respectively, and Sejen et al. also reported the use of the PLGA (LA : GA : 50 : 50) [57]. The DSC analysis showed only the Tg transition and did not show any molten thermal peak, indicating that the electrospun PLGA nanofibers showed a non-crystalline structure or amorphous character [33,56].

The amorphous phase was verified by XRD analysis (Figure 7B), where the PLGA and PLGA/collagen showed a wide peak characteristic of the random polymer structure [57]. Similar results were reported by Mollaeva et al., where the PLGA displayed a dome-shaped region between 10 to 30° due to an amorphous state [58].

On the other hand, the lyophilized collagen thermogram shows that the denaturation temperature occurs at 98 °C. The Td is the temperature at which the collagen loses its ordered triple helical structure to form a random coil and is directly associated with the evaporation initial of bonded water responsible for the stability of the triple helix conformation of collagen macromolecules [59]. Therefore, Td can be considered a measure of collagen’s thermal stability as it gives the temperature for unfolding on heat treatment [60]. In the thermogram curve of the PLGA/collagen scaffold, this Td for collagen was not observed, indicating that there is no loss of the structural nature during the electrospinning process, as was observed previously by FTIR.

Moreover, the thermogram profile for PLGA/collagen was similar to that of PLGA fibers, suggesting a miscible blend and adequate integration of collagen into the fibers during the processing method of the scaffolds. The Tg value for PLGA in the PLGA/collagen did not present a change, indicating that the incorporation of collagen does not affect the transition of the glassy to a rubbery state of the PLGA Chakrapani et al. prepared nanofibres from polycaprolactone (PCL) with type I collagen blends by electrospinning. They observed no significatively changes in the Tg of PCL under the incorporation of collagen, and the Td of collagen was masking in the thermal profile due to a more significant relation of PCL even in the presence of lower amounts of collagen [61].

Furthermore, a thermogram of collagen prepared from solvent casting in HFIP was collected in order to understand the thermal behavior of type 1 collagen when it was in the presence of the solvent. Figure 7A indicates an influence of the solvent despite its evaporation, and in this case, the Td of the collagen is shifted to a temperature ca. 124 °C, which indicates a maximization of collagen-collagen interactions due to the presence of interchain hydrogen bonds [62]. The stabilization of the native collagen after contact with HFIP confirms that the solvent does not affect its structural nature and is not conducive to its denaturalization. This was also confirmed by other authors who have shown that the structure of electrospun collagen using solvents such as HFIP did not considerably affect collagen’s helical structure [36].

### 3.6. Mechanical Properties

**Figure 8:**
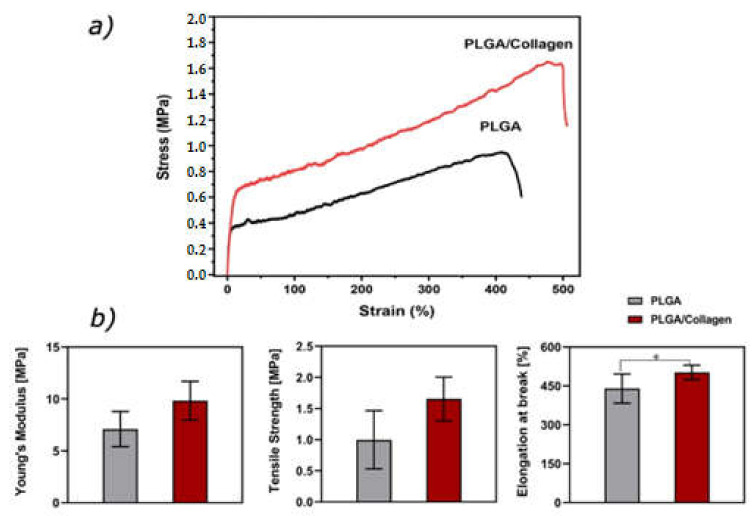
Mechanical analysis of PLGA and PLGA/collagen electrospun fibers (**a**). Strain-Stress curve and (**b**) Statical analysis of Young’s modulus, Tensile strength, and elongation at break of PLGA and PLGA/collagen scaffolds. (* denotes a significant difference with a value *p* < 0.05) (*n* = 6).

The mechanical behavior of biomaterials is an important parameter in order to know their possible applications projecting their use in physiological conditions. In this sense, the PLGA and PLGA/collagen electrospun fibers were characterized by uniaxial tensile testing, the mechanical properties of both scaffolds are shown in Figure 8. It is possible to observe the values of Young’s modulus, tensile strength, and elongation at break for both fibers’ mats prepared. The values of Young’s modulus for PLGA and PLGA/collagen were 7.10 ± 1.7 and 9.8 ± 1.9 MPa, respectively. On the other side, these results are concordant with the increase of tensile strength that reaches values close to 1.0 ± 0.3 and 1.7 ± 0.4 MPa for PLGA and PLGA/collagen, indicating that the incorporation of collagen improved the stiffness of the material. This phenomenon is more clearly appreciated in the Strain-Stress curve (Figure 8a).

Furthermore, the statistical analysis showed that only the elongation at break showed a significative difference (*p* < 0.005) (Figure 8b). Our results indicate that collagen incorporation increases the value of Young’s modulus and tensile strength. However, this variation is not significant. José et al. prepared fibers based on blends of PLGA/collagen (type I) in ratios (80:20), (65:35), and (50:50), finding that the incorporation of collagen decreases the elastic modulus and tensile strength. On the other side, this effect is more considerable when the amount of collagen is higher [30]. Considering the results obtained and the low incorporation of collagen (2 wt%) in the PLGA matrix, the increase in mechanical properties could be mainly associated with the decrease in fiber size. Kim et al. and Morel et al. reported that the fiber diameter plays a preponderant role in mechanical behavior, concluding that when the fiber diameter decreases, increase the stiffness of the material showed an increase in the values of Young’s modulus and tensile strength. This phenomenon is associated with the fact that the reduction in the fiber’s size increases the fibers’ stronger orientation in the amorphous phase, as well as a higher fraction of oriented polymeric chains within thinner fibers, which gives them greater rigidity [63,64].

### 3.7. Cell Adhesion and Viability

**Figure 9:**
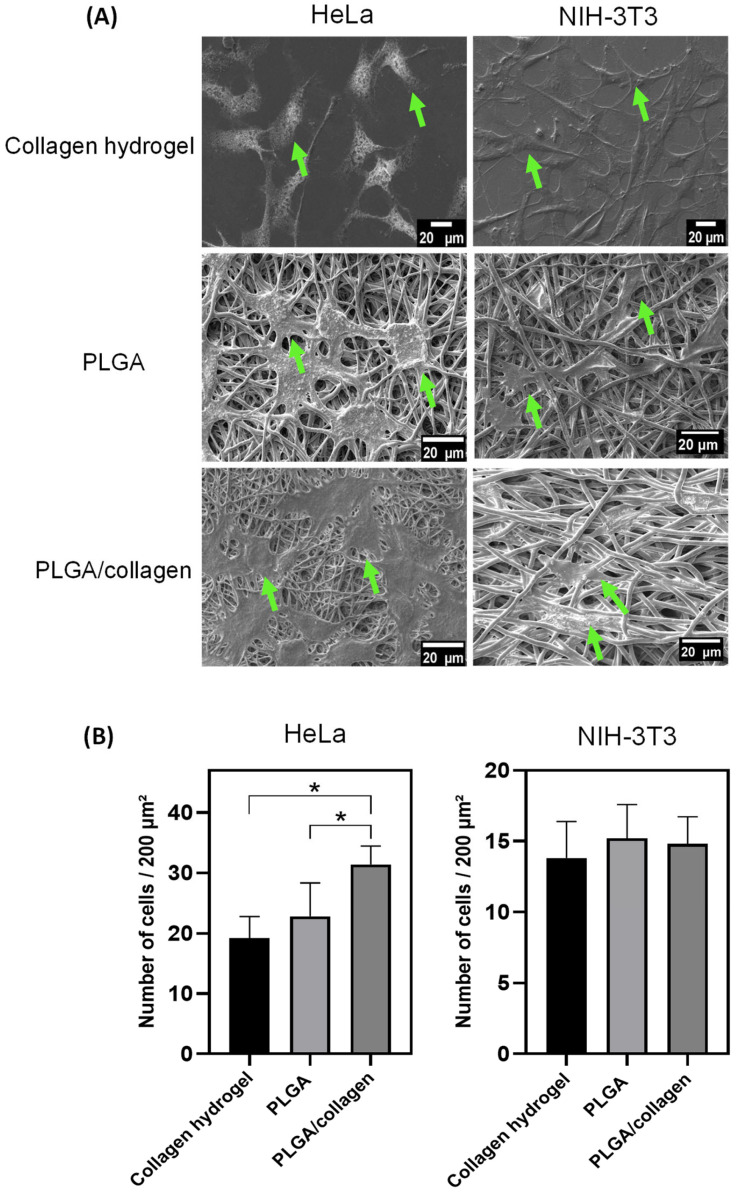
(**A**) SEM images of HeLa cells and NIH 3T3 attached to the collagen hydrogel, PLGA, and PLGA/collagen scaffolds for 48 h. (**B**) The number of HeLa cells and NIH 3T3 adhered to scaffolds. (* denotes a significant difference with a value *p* < 0.05, and arrows indicate the presence of cells on scaffolds).

**Figure 10:**
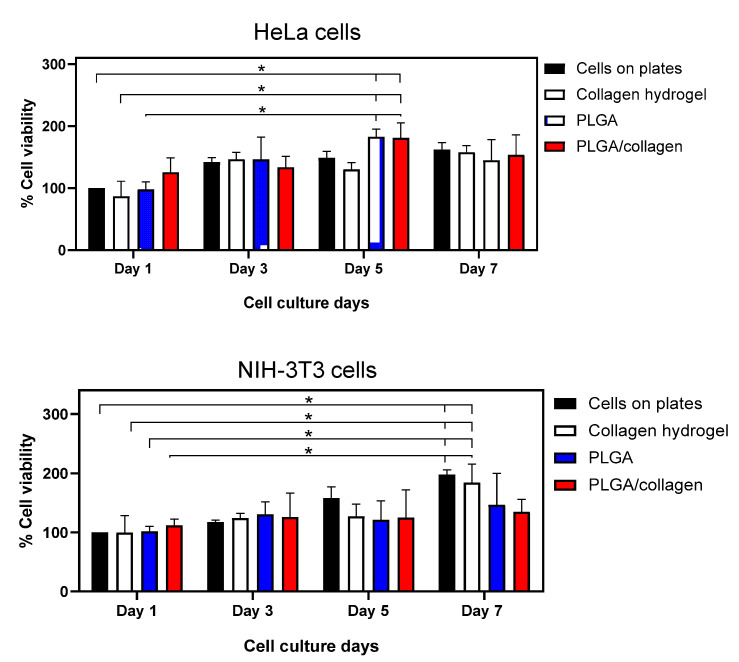
MTT test of cell viability using HeLa cells and NIH-3T3 fibroblasts on a positive control, collagen hydrogel, PLGA, and PLGA/collagen scaffolds at different exposure times, 1, 3, 5, and 7 days. (* denotes a significant difference with a value *p* < 0.05).

Electrospun scaffolds must allow proper adhesion and cell proliferation so that cells at the time of adhering to the scaffold can regulate the cell signaling process that favors tissue regeneration [60]. The adhesion cell and the percentage of cell viability of the scaffolds with HeLa cells and NIH-3T3 fibroblasts are shown in Figure 9 and Figure 10. For comparison purposes, a positive control based on only cells on plates and collagen hydrogel as a 3D control were used. As seen in the SEM images (Figure 9A), HeLa with a polygonal epithelial morphology and NIH-3T3 cells with elongated and fusiform morphology were spread along the collagen hydrogel and randomly on fibers after 48 h, indicating that both cell lines were adequately attached in the surface of the scaffold. PLGA/collagen scaffold presented a significant increment in the HeLa cell amount (31 ± 3.05 cells/200 μm^2^) compared to the PLGA scaffold (23 ± 5.54/200 μm^2^) and control of collagen hydrogel (19 ± 3.56 cells/200 μm^2^) while the number of NIH-3T3 cells adhered was similar in all scaffolds with a value ca. 15 ± 2.38/200 μm^2^ (Figure 9B). Figure 10 shows that HeLa cell viability was maintained mainly until day 5 in the PLGA and PLGA/collagen scaffolds compared to collagen hydrogel, which showed significantly lower cell growth. In contrast, a significant increase of NIH-3T3 cells was maintained until day 7, but the cell viability of the scaffolds was lower than that of both controls. These results demonstrated that the porous and interconnected 3D morphology of the scaffold provided a structural design with capacity, suitability, and a favorable microenvironment for hosting cells. The cell adhesion and growth in the fibrous matrix have been associated with the presence of collagen, which is known to enhance scaffolds’ biocompatibility [65,66] due to its interaction with the cells through discoidin domain receptors (DDRs) and integrin α1β1 [67,68].

On the other hand, Chen et al., 2020, studied the adhesion and proliferation of hMSCs cells on highly porous PLGA/collagen scaffolds. With only 0.5% (*w*/*v*) type, I collagen incorporated into the scaffold, hMSCs were adhered and with extended cell morphology after 1 day of culture and, after 7 days, presented the highest proliferation on scaffolds. The authors mentioned that the good cell adhesion cell was due to the interaction between the RGD sequence of collagen in the scaffold and the αvβ3 integrin receptors in hMSCs [69,70]. The cell-scaffold interaction depends not only on the collagen matrix but also on various properties associated with the morphology of the fibrous scaffolds, for instance, the effect of fiber and pore size, considering that both parameters presented changes in this study for PLGA/collagen compared to PLGA. Furthermore, exists a direct correlation between the fiber diameter and cell behavior; this means that a decrease in the diameter of the fiber and larger porosities are directly related to the increase in the adhesion cell, attributing this effect to an increase in the specific surface area, which enhances the protein absorption [71,72,73]. Asaga et al. have proposed that the interaction between cell-collagen also depends on the cell surface groups, as is the case of the glycoproteins in fibroblast, influencing the way cells are adhered to and probably proliferate on the surface of the scaffolds [74].

Another parameter that can influence cell adhesion and proliferation is the change in the mechanical properties of the scaffolds. Other authors explained that better mechanical support for cell growth is directly related to higher mechanical strength. For instance, Baker et al. explained that the closer storage modulus of PCL scaffolds to the native tissue value could increase cell viability [75], while Pauly et al. indicated that an increase in the mechanical properties in PCL scaffolds (Young’s modulus of 2.4 MPa) had higher cell growth [76]. Our results indicated that the porous and fibrillar structure of scaffolds obtained by electrospinning mimicking the ECM and the increase in mechanical strength of PLGA/collagen scaffold could positively affect the biological performance, favor the affinity, and improve the cell adhesion and viability after 7 days.

### 3.8. Collagen Release Assay

**Figure 11:**
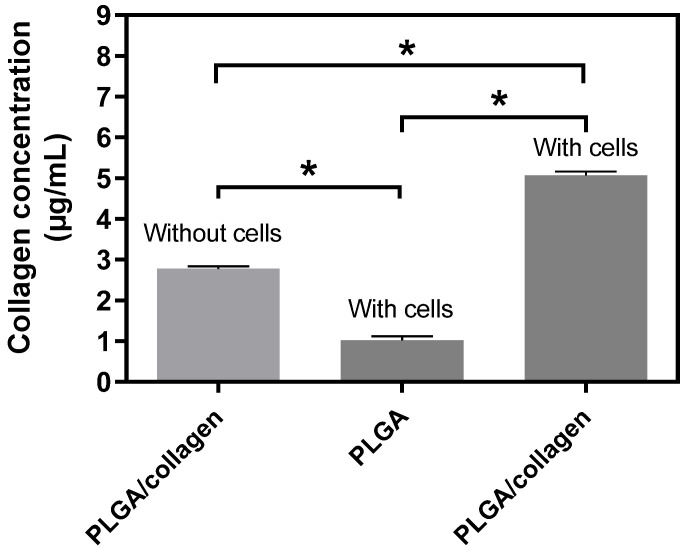
Collagen release by NIH-3T3 fibroblasts cultured on PLGA and PLGA/collagen scaffolds. As a control group, PLGA/collagen scaffolds without cells were incubated. (* denotes a significant difference with a value *p* < 0.05).

With the purpose of exploring whether the PLGA/collagen scaffolds could be useful as a support for ECM regeneration, here we determined the effect of these scaffolds on the release of collagen in NIH-3T3 fibroblasts (Figure 11). We found that PLGA/collagen scaffolds released significantly more collagen (5.06 ± 0.15 μg/mL) compared with PLGA scaffolds in contact with fibroblasts (1.01 ± 0.18 μg/mL) or PLGA/collagen without cells (2.77 ± 0.10 μg/mL). The level of collagen in the group PLGA/collagen without cells was higher than in PLGA scaffolds with cells, which could be explained by a spontaneous release of collagen from the PLGA/collagen scaffolds. However, since PLGA/collagen scaffolds incubated with cells had a higher collagen level than those incubated without cells, we can assume that PLGA/collagen fibers promote increased collagen release in NIH-3T3, suggesting that our biomaterial could induce ECM neoformation. Fibroblasts are the main inductors of ECM regeneration, inducing the secretion of type 1 collagen, which is the main component of the extracellular matrix and contributes to the resistance of tissues and generates biological signals that contribute to tissue repair. Since fibroblasts express collagen, it is important to stimulate these cells to generate an improvement in tissue regeneration [77]. We postulate that the excellent mechanical properties of PLGA/collagen scaffolds compared to PLGA scaffolds help to increase collagen gene expression, stretching the fibroblasts to bind with integrins and provide resistance to the contractile force of the cytoskeleton [78]. Thus, the maintenance of the structure and function of the extracellular matrix could be supported by the PLGA/scaffolds, reinforcing our proposition that these scaffolds could be useful in tissue bioengineering therapy. On the other hand, there are important growth factors and molecules such as TGFβ1 and integrins in tissue repair, which regulate the processes of cell proliferation and production of new extracellular matrix, including collagen [79]. Probably, the PLGA/collagen stimulates the secretion of some of these molecules, but this remains to be elucidated.

## 4. Conclusions

In the present study, scaffolds with a fibrillar, porous, and interconnected structure were designed by electrospinning from a blend of (PLGA) and collagen to be applied as adequate candidates for tissue engineering. The random fiber diameter decreased in the presence of collagen compared to PLGA due to a change in the conductivity solution in the electrospinning process. It was founded by IR and thermal analysis that collagen presents structural stability in the blend and then was subjected to the electrospinning process. Scaffolds presented resistance to the enzymatic degradation process for 1 week in the presence of collagenase, indicating that it can favor tissue regeneration processes in a short period of time. Besides, the rigidity of the PLGA was affected by the presence of collagen, and the elastic modulus and tensile strength increased for PLGA/collagen compared to PLGA.

The presence of collagen in the fibers provided a suitable environment for the cell adhesion and viability of human cervical carcinoma HeLa and NIH-3T3 fibroblast of PLGA scaffolds during 7 days due to the good interaction between collagen and cells. Besides, fibroblast cells promoted collagen secretion from scaffolds, indicating that PLGA/collagen scaffolds could induce ECM neoformation. Our results also show that the fibrillar and porous morphology positively affected the biological performance of both PLGA and PLGA/collagen scaffolds, suggesting that this material could be an adequate support to stimulate extracellular matrix remodeling for a potential application in engineering tissue.

## Data Availability

Not applicable.
